# Preparation and characterization of B_4_C coatings for advanced research light sources

**DOI:** 10.1107/S1600577515020901

**Published:** 2016-01-01

**Authors:** Michael Störmer, Frank Siewert, Harald Sinn

**Affiliations:** a Institute of Materials Research, Helmholtz-Zentrum Geesthacht, Max-Planck-Strasse 1, D-21502 Geesthacht, Germany; b Institute for Nanometre Optics and Technology, Helmholtz-Zentrum Berlin, Albert-Einstein-Strasse 15, 12489 Berlin, Germany; c European XFEL GmbH, Albert-Einstein-Ring 19, 22761 Hamburg, Germany

**Keywords:** FEL, X-ray optics, coatings, mirrors

## Abstract

X-ray optical elements are required for beam transport at the current and upcoming free-electron lasers and synchrotron sources. An X-ray mirror is a combination of a substrate and a coating. The demand for large mirrors with single layers consisting of light or heavy elements has increased during the last few decades; surface finishing technology is currently able to process mirror lengths up to 1 m with microroughness at the sub-nanometre level. Additionally, thin-film fabrication is able to deposit a suitable single-layer material, such as boron carbide (B_4_C), some tens of nanometres thick. After deposition, the mirror should provide excellent X-ray optical properties with respect to coating thickness errors, microroughness values and slope errors; thereby enabling the mirror to transport the X-ray beam with high reflectivity, high beam flux and an undistorted wavefront to an experimental station. At the European XFEL, the technical specifications of the future mirrors are extraordinarily challenging. The acceptable shape error of the mirrors is below 2 nm along the whole length of 1 m. At the Helmholtz-Zentrum Geesthacht (HZG), amorphous layers of boron carbide with thicknesses in the range 30–60 nm were fabricated using the HZG sputtering facility, which is able to cover areas up to 1500 mm long by 120 mm wide in one step using rectangular B_4_C sputtering targets. The available deposition area is suitable for the specified X-ray mirror dimensions of upcoming advanced research light sources such as the European XFEL. The coatings produced were investigated by means of X-ray reflectometry and interference microscopy. The experimental results for the B_4_C layers are discussed according to thickness uniformity, density, microroughness and thermal stability. The variation of layer thickness in the tangential and sagittal directions was investigated in order to estimate the achieved level of uniformity over the whole deposition area, which is considerably larger than the optical area of a mirror. A waisted mask was positioned during deposition between the sputtering source and substrate to improve the thickness uniformity; particularly to prevent the formation a convex film shape in the sagittal direction. Additionally the inclination of the substrate was varied to change the layer uniformity in order to optimize the position of the mirror quality deposited area during deposition. The level of mirror microroughness was investigated for different substrates before and after deposition of a single layer of B_4_C. The thermal stability of the B_4_C layers on the various substrate materials was investigated.

## Abstract 2

The challenging specifications for long X-ray mirrors for upcoming free-electron lasers can be achieved, especially for maintaining below 2 nm peak-to-valley shape error along the optical aperture of approximately 1 m-long mirrors.

## 1. Introduction   

Long X-ray mirrors are required at advanced research light sources in order to transport or shape the photon beam. The demands on surface quality and finishing of such mirrors are extremely high at third-generation storage-ring-based X-ray sources. The mirrors are used as standard components for collimating, focusing and low-pass filtering (US DOE, 2013 ▸). At new diffraction-limited X-ray sources such as free-electron lasers (FEL) or ultimate storage rings, the demands on beamline optics will be even more challenging. In particular, worldwide there are VUV and hard X-ray FEL sources under operation such as FLASH (Germany) (Tiedtke *et al.*, 2009 ▸), LCLS at SLAC (USA) (Boutet & Williams, 2010 ▸), FERMI at ELETTRA (Italy) (Cocco *et al.*, 2010 ▸) and SACLA (Japan) (Ishikawa *et al.*, 2012 ▸). Other projects are planned such as the SwissFEL at PSI (Switzerland) (Patterson *et al.*, 2010 ▸). Another facility currently under construction is the European XFEL (Germany) (Altarelli, 2011 ▸), which will deliver FEL radiation *via* three different beamlines (two hard X-ray beamlines operating at 3–24 keV and one soft X-ray beamline operating at 0.26–3 keV) to six experimental stations. A variety of X-ray optical elements, such as mirrors and gratings (Vannoni *et al.*, 2013 ▸), are required for FEL beam transport to various experiments which will exploit the high intensity, coherence and time structure of the new source. X-ray mirrors are located at a large distance from the source; thus, the beam size can increase and the enormous peak power can be reduced to avoid single-shot damage (Chalupský *et al.*, 2009 ▸). The distance of 250–300 m from the FEL source to the first mirror is a compromise between the reduction of photon fluence and the available length of ultra-precision mirrors. Operating the mirrors at large distances and well below the critical angle significantly reduces the risk of beam damage. However, single-shot damage (Nüske *et al.*, 2011 ▸) and thermally induced effects (Dastjani Farahani *et al.*, 2011 ▸) in coated mirrors are ongoing fields of research and investigation.

It is worthwhile mentioning that the source size and the source distance determine the acceptable slope error. A source size of 20 µm r.m.s. viewed at a mirror distance of, for example, 1000 m smears out the incident angle on a mirror surface by only 20 nrad r.m.s., which can be viewed as a maximum tolerable slope error for the mirror, if the beam should remain diffraction limited. Therefore, the technical requirements of X-ray mirrors are a challenge in view of both substrate finishing and coating technologies. Metrology measurement methods are currently at their limits, especially for determining the radius of the upcoming X-ray mirrors required for the European XFEL project (Sinn *et al.*, 2011 ▸) in Hamburg (Germany). In this case a radius of curvature of more than 6000 km is required and needs to be measured. Furthermore, it is a challenge for coating technology to deposit single layers with high uniformity over a distance of more than 1 m.

The technical specifications are extremely tight, especially the requirement that the shape error of the mirror be less than 2 nm peak-to-valley (PV) over the entire optical area. The achievable limits of substrate fabrication are being continuously improved to establish very long and ultra-smooth substrates with low microroughness in various spatial frequency ranges (Siewert, 2013 ▸). The substrate finish of such high-quality substrates over a length of 1 m can be improved by ensuring contamination-free deposition conditions. The mirror reflectivity and its resistivity towards single-shot damage are improved by both reduced surface roughness and contamination of the substrate or coating. After surface finishing, a substrate is coated with a suitable film to change its surface according to the X-ray optical requirements in terms of an optimal photon flux for the desired application. The fabricated X-ray mirror (*i.e.* substrate and coating) should maintain important properties such as the above-mentioned low roughness that are predetermined by the surface of the substrate. One requirement is that the coating process does not increase surface roughness or alter the surface morphology in such a way that the reflectivity of the mirror is reduced. The coating material on top of a highly polished substrate can be selected according to the required grazing incidence angle for total reflection. The critical angle is determined by photon energy and the density of the coating material. The combination of magnetron-sputtered amorphous carbon (a-C) on a silicon substrate works reliably in the soft X-ray range at FLASH (Tiedtke *et al.*, 2009 ▸; Störmer *et al.*, 2010 ▸). For hard X-rays, a promising coating material is boron carbide (B_4_C) because it contains light elements and has a high melting point of about 2400°C (Thévenot, 1990 ▸).

The bulk properties of ceramic B_4_C are very interesting because it is the third hardest material after diamond and cubic boron nitride (Kokai *et al.*, 2001 ▸). This refractory material is also of interest for body armour (Fanchini *et al.*, 2006 ▸). It is a *p*-type semiconductor and, in addition to outstanding hardness, it also possesses high resistance to chemical reagents (Thévenot, 1990 ▸). Magnetron-sputtered films of B_4_C were prepared and characterized after the development of high-density sputtering targets of B_4_C (McKernan, 1991 ▸). Various sputtering parameters were investigated in order to alter the thin-film properties with regard to hardness and internal stress (Wu *et al.*, 2004 ▸). Various techniques have been used for thin-film preparation, *e.g.* laser deposition (Kokai *et al.*, 2001 ▸; Aoqui *et al.*, 2002 ▸). The sputtered films of B_4_C exhibited compressive stresses and high hardnesses, which are desired properties for cutting tools (Jagannadham *et al.*, 2009 ▸). Super-hard coatings with hardness values of about 70 GPa were achieved by ion-beam sputtering (Ulrich *et al.*, 1998 ▸). For X-ray optical requirements, the morphology, microstructure, stress and damage were studied in order to apply the coatings for FEL applications (Soufli, Pivovaroff *et al.*, 2008 ▸, 2009 ▸; McCarville *et al.*, 2008 ▸; Barty *et al.*, 2009 ▸). The optical constants of B_4_C coatings were investigated in the soft X-ray range (Soufli, Aquila *et al.*, 2008 ▸).

Radiation stability is an important issue for the use of B_4_C-coated mirrors. Many investigations have been performed to evaluate this promising material. X-ray ablation experiments of eight materials (B_4_C, B, SiC, C, SiN, Al, Al_2_O_3_ and SiO_2_) relevant to the target chamber design of the National Ignition Facility were performed and compared with a transient ablation model (Anderson *et al.*, 1996 ▸). The two goals were to predict material removal due to X-rays and to determine the state of the ablated material. That work clearly demonstrated a high resistance of boron carbide to nanosecond-long pulses of keV radiation. Irreversible damage to the B_4_C/W multilayer mirrors was observed after soft X-ray laser pulses (Le Guern *et al.*; 1996 ▸). After two pulses (fluence of 0.05 J cm^−2^ per shot and pulse duration of 1 ns), multilayer reflectivity decreased and the period was expanded by 5% with respect to the unirradiated mirror. The interaction of X-ray FEL pulses with boron carbide was studied at LCLS (Hau-Riege *et al.*, 2010 ▸) and SACLA (Aquila *et al.*, 2015 ▸). The earlier of these articles described the exposure of bulk B_4_C and thin SiC films, and the latter the fluence thresholds of grazing-incidence hard X-ray mirrors coated with thin layers of ruthenium and boron carbide.

This article presents our latest thin-film developments relating to B_4_C coatings with a length of up to 1500 mm and a width of 120 mm. The challenge is to repeatably manufacture stable and highly uniform coatings, which are essential for the application of the XFEL mirrors. This must be done without changing the surface properties, which are predetermined by ultra-precise substrates. The HZG sputtering facility will be used to coat worldwide unique silicon substrates with extraordinarily high technical specifications, particularly the shape error of the blanks which will be below 2 nm PV along the long mirror axis of 1000 mm. After coating, these mirrors will be used in the 3.4 km-long tunnel system of the upcoming European XFEL to transport the beam to the experimental hutches. It is expected that the coatings, especially the B_4_C layers, will not change the shape error of the mirror. It should be appreciated that it is very challenging to investigate thin-film properties such as layer uniformity, microroughness and thermal stability. The precision, stability and repeatability of the sputtering facility have been extensively investigated so that further advances can be made in coating technology and the manufacture of outstanding X-ray optics for advanced research light sources.

## 2. Experimental   

The single layers were fabricated on test substrates using the 4.5 m-long HZG magnetron sputtering facility that has a deposition length of 1500 mm and a width of 120 mm. The ultra-high vacuum chamber is comprised a load lock and a deposition chamber that are evacuated by a turbomolecular pump and a cryo-pump. A laminar flow box for the pre-treatment and cleaning of the well polished silicon (20 mm × 60 mm × 0.65 mm) and Al_2_O_3_ (sapphire) substrates (10 mm × 10 mm × 0.53 mm) is located in front of the load lock flange. Typical parameters of the deposition chamber are a base pressure of less than 1 × 10^−5^ Pa, a source–substrate distance of about 12 cm and an argon gas pressure of 0.12 Pa. The generator power is 800 W MF (mid-frequency). A rectangular 88.9 mm × 355 mm magnetron sputtering source was used to coat the small test substrates that were fixed on a large mirror dummy that was movable along the tangential (*x*) direction. In the case of the single layers, the sagittal (*y*) direction, which is perpendicular to the tangential direction, was also investigated by means of X-ray reflectometry (XRR). Sixteen small test substrates were placed along the centre of the mirror dummy (the *x* direction). Additionally two test substrates, offset from the centre-line were placed at nine positions along the mirror dummy length. Taken together 34 test substrates were used to simulate a total area of 1500 mm × 120 mm, which is the maximum available deposition area. The whole configuration of 34 substrates will be referred to as a single-layer mirror in the following text. For the characterization of a multilayer mirror (Siewert *et al.*, 2014 ▸), an area 500 mm long consisting of an array of eight substrates was sufficient for investigating the tangential direction. This is because in application a multilayer mirror can be shorter than a single-layer mirror, for example a double-multilayer mirror system for an offset in a beamline.

The single layers were investigated by means of X-ray reflectometry using Cu radiation (X-ray wavelength of 0.154 nm). A D8 Advance (Bruker) diffractometer equipped with a reflectometry stage and a knife edge was used. A Göbel mirror behind the source was employed to form a parallel and monochromatic beam (Störmer *et al.*, 2007 ▸). All XRR measurements were performed on short calibration samples. The reflectivity scans were analysed with *REFSIM* and *LEPTOS R* (Bruker) simulation software. Some selected scans were also fitted using David Windt’s *IMD* software (Windt, 1998 ▸).

Microroughness measurements were performed using atomic force microscopy (AFM) and white-light interferometry at the Helmholtz-Zentrum Berlin (HZB). Atomic force microscopy is a scanning probe microscopy technique providing height resolution on the atomic scale (Binnig *et al.*, 1986 ▸; Meyer, 1992 ▸). It is a surface-sensitive method that probes real-space lengths and is complementary to X-ray scattering. The atomic force microscope used was a Bruker SIS-Ultra-objective with a 40 µm × 40 µm scanner and was located on a PICO-station system with active vibration damping. The tip used for these measurements in the non-contact mode was a silicon SPM-sensor with a resonance frequency of 190 kHz and force constant of 48 N m^−1^. The tip had a height of 10–15 µm and a radius of less than 8 nm. Thus, the achievable lateral resolution is about 10 nm. After 10 scans the tip was changed to avoid measurements being influenced by the wearing of the tip. White-light interferometry, as an alternative method with higher spatial frequency, was also used to measure the microroughness of three coatings and uncoated silicon substrates. A Micromap Promap 512 white-light interferometer was used with Mirau-type interferometer objectives studying microroughness on a spatial wavelength range from 235.2 µm to 1.7 µm (magnification of 20×) and from 94 µm to 0.62 µm (magnification of 50×).

## 3. Reflectivity measurements and thickness uniformity of magnetron-sputtered B_4_C coatings   

**Figure 1:**
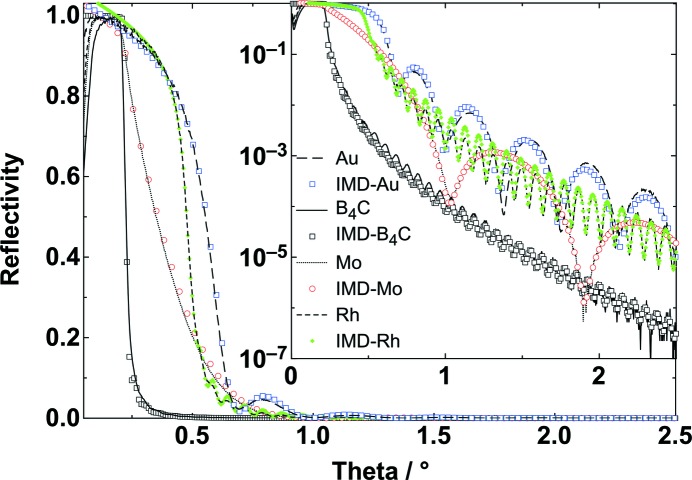
Specular X-ray reflectivity as a function of the incidence angle for Au, B_4_C, Mo and Rh coatings. The scans were performed using Cu radiation (8048 eV). The results from *IMD* simulations are also indicated. The linear plot shows that the critical angle is higher when the film density is increased. The logarithmic scale of the insert depicts thickness oscillations (Kiessig oscillations). The distance between the maxima is smaller for thicker films.

Specular reflectivity scans comparing a boron carbide coating to some alternative mirror coating materials are shown as a function of the incidence angle in Fig. 1 ▸. The four measurements were performed with Cu radiation (8048 eV) over 2.5° with a step size of 0.003°. The density of the measuring points in the figure is reduced for clarity. The difference in critical angle is clearly visible in the linear plot. The logarithmic insert demonstrates Kiessig oscillations and their change in frequency, which is determined by the layer thickness of the mirror coating material. The selected materials cover a broad range of densities. The boron carbide, molybdenum, rhodium and gold films have layer thicknesses of 58.8, 4.8, 45.3 and 10.7 nm, respectively. The coating properties determined using IMD simulations are shown in Table 1 ▸. The scans for the four coating materials are listed according to their density. The critical angles were also obtained using the *IMD* software package. As expected, the thin layers of Rh and Au exhibit higher critical angles of 0.47 and 0.55°, respectively, due to their high densities. The measured critical angle of Mo is about 0.35°; this indicates that its thickness (4.8 nm) is too small to reach the expected critical angle of 0.43° for mid photon energy used (Henke *et al.*, 1993 ▸). Thus, the measured reflectance of the Mo single layer becomes rounded in the region of total external reflection due to transmittance of the very thin layer (VDI/VDE, 2011 ▸). The reflectivity measurements for the B_4_C coating give a critical angle of about 0.22° (*i.e.* 3.9 mrad) for a film density of about 2.37 g cm^−3^. This is quite a high value at about 94% of the bulk density of 2.52 g cm^−3^ (Thévenot, 1990 ▸). This is in agreement with previous results (Störmer *et al.*, 2010 ▸, 2011 ▸; Kozhevnikov *et al.*, 2015 ▸). In comparison with magnetron sputtering, other thin-film methods are not able to achieve such a high density. For instance, films prepared by chemical vapour deposition exhibit a reduced density owing to the incorporation of hydrogen during deposition (Oliveira & Conde, 1997 ▸).

For X-ray investigation of thin films using Cu radiation, it is worthwhile mentioning that the native silicon oxide layer on top of the silicon substrate enables a more accurate determination of the thickness and other coating properties as it differentiates the position between the single layer of boron carbide and the substrate which have only a small difference in density. The measured specular Cu *K*_α_ reflectivity (8048 eV) of a magnetron-sputtered B_4_C layer is above 90% at incidence angles below 0.21° (*i.e.* 3.7 mrad).

**Figure 2:**
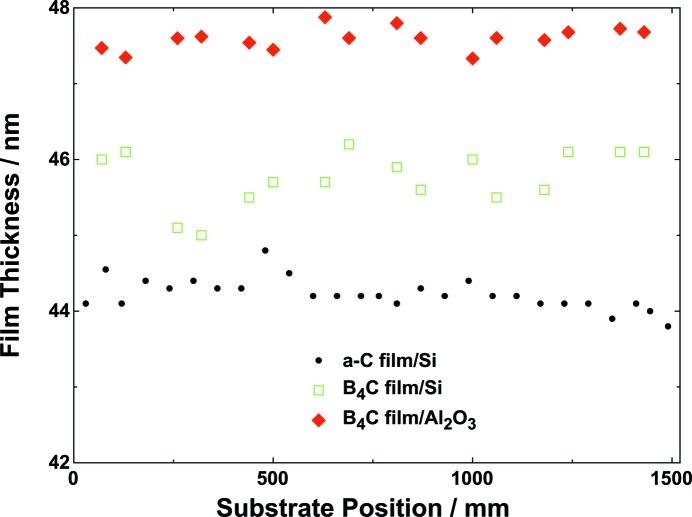
B_4_C coating layer thickness in the tangential direction (*x*, which is parallel to the long axis) of the mirror.

The variations in layer thickness in the tangential direction for three different combinations of single-layer materials and substrates are shown in Fig. 2 ▸. The experimental data for a-C on Si and for B_4_C on silicon and on sapphire substrates are shown as circles, squares and diamonds, respectively. The variation in film thickness as a function of the *x* position was determined to give a measure of the tangential thickness uniformity. The mean thickness of the a-C/Si, B_4_C/Si and B_4_C/Al_2_O_3_ single layers were determined to be 44.2, 45.6 and 47.6 nm. The related PV values were 1.0, 1.6 and 0.5 nm, respectively. The percentage variations in thickness over the whole 1500 mm length were 2.3, 3.5 and 1.1% when compared with the mean thickness. The difference in B_4_C thickness coatings on the Si and Al_2_O_3_ substrates probably results from differences in the density of the substrate materials. The film density of the magnetron-sputtered B_4_C layers (2.37 g cm^−3^) is very similar to the bulk value for silicon (2.33 g cm^−3^) but is quite different to that of sapphire (3.99 g cm^−3^). A higher difference in density between the deposited layer and substrate improves the accuracy of the thickness measurements. The experimentally determined thickness variation is very close to the typical limit of ±1%, which results from insufficient mechanical stability during thin-film preparation (Ohring, 2002 ▸).

**Figure 3:**
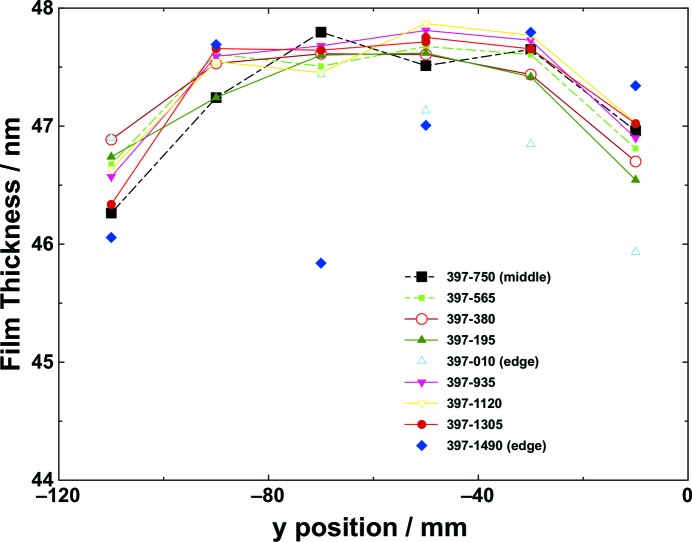
B_4_C coating layer thickness in the sagittal direction (*y*, which is perpendicular to the long axis) of the mirror.

The film thickness was also measured in the sagittal direction (*y*) of the mirror (see Fig. 3 ▸). The layer thickness of B_4_C decreases by up to 5% towards the outer deposition area, which is mainly caused by the limited sputtering profile of the source. A waisted mask is used in front of the deposition source in order to compensate and flatten the thickness distribution in the sagittal direction. Over a 120 mm distance, the PV value is about 2.0 nm, which corresponds to a circle with a radius of 960 km, *i.e* ≃1/7 of the radius of the earth. It is important to be able to measure these exceptional values accurately for upcoming advanced light sources. Some imperfections are visible and are most likely due to shadowing effects along the edges of the long mirror axis at *x* = 10 mm and 1490 mm. For future X-ray FEL mirrors, the optical area will be smaller than the available area on the carrier, which will be discussed below. It is important to mention here that further improvement of the sagittal thickness variation should be possible with a more appropriate mask.

**Figure 4:**
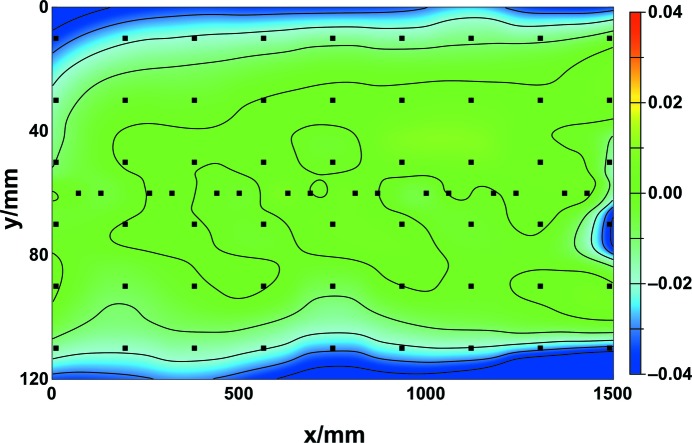
Uniformity of the B_4_C film layer thickness. The coating was deposited on 70 sapphire substrates, which are evenly disributed over the available area of 1500 mm (tangential) by 120 mm (sagittal). The coloured *z*-scale is the normalized layer thickness difference given by (*t*−*t*_m_)/*t*_m_, where *t*_m_ is the mean layer thickness along the tangential direction (16 positions, *y* = 60 mm) and *t* is the measured thickness at 70 positions within the mirror area (*x*, *y*). Contour lines have been interpolated in order to demonstrate the changes. The values of normalized difference decrease towards the outer regions of the area.

The specular X-ray reflectance of B_4_C layers on sapphire test substrates, placed on a mirror dummy, was measured at 70 positions in order to investigate a prospective large optical area of 1500 mm × 120 mm. The sapphire test substrates were positioned on the mirror dummy in the same array as described earlier for the silicon test substrates. In total 70 scans were analysed in order to determine the B_4_C layer thickness and to build a contour plot representative of a possible large-area mirror (Fig. 4 ▸). The mean thickness in the tangential direction was 47.6 nm with a PV value of 0.5 nm as described above. Over the whole area, the mean thickness was similar at 47.3 nm and the PV value increased slightly to 2.0 nm. The thickness at the middle of the coated area was higher than at the edges due to the deposition profile of the magnetron source as mentioned above. This profile was successfully modified using a waisted mask, similar to that which was first developed for deposition of amorphous carbon (Störmer *et al.*, 2010 ▸), during the deposition process. However, the uniformity of the B_4_C layer thickness is still slightly peaked with minor asymmetries. The uniformity could be further enhanced with a more restrictive mask, which is under current development. Without doubt the experimental results achieved are fully acceptable for current thin-film fabrication of FEL mirrors. A typical mirror for the upcoming European XFEL has a length of 800 mm and a width of 80 mm. During mirror production the substrate will be centred within the available deposition area in order to achieve a PV value of less than 0.7 nm. It is worth noting that the deposition length at HZG can be increased to 1300 mm without compromising the excellent PV value.

**Figure 5:**
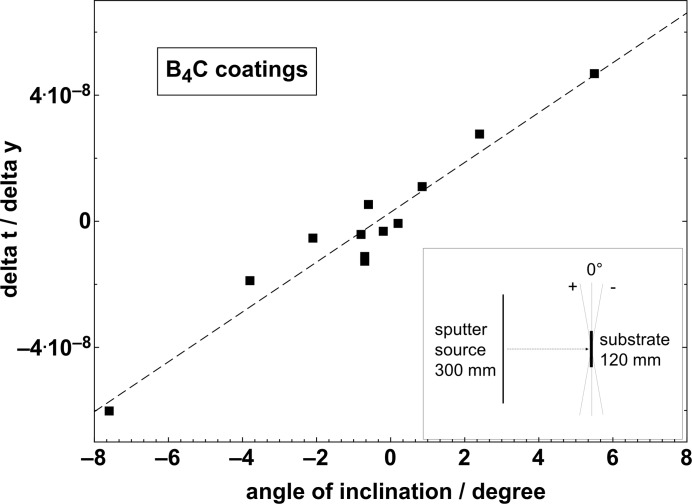
Ratio (*m*) of the difference in thickness to difference in *y* position as a function of the inclination angle (insert: geometrical dimensions between the sputtering source and substrate).

The inclination of the substrate during the deposition process is very important in order to deposit a uniform coating in the sub-nanometre thickness range over a large optical area. The inset in Fig. 5 ▸ demonstrates the geometrical set-up and the three most important operating distances: 300 mm-long sputtering source, 120 mm-long substrates and a source–substrate distance of about 120 mm. The inclination is the angle subtended between the line of shortest distance from the substrate to the source and the normal to the optical plane of the mirror. Each filled square in Fig. 5 ▸ represents a coating prepared under a fixed incidence angle. The angle was varied over a range of ±8° and with a smaller step size near to the vertical position (0°). The vertical position is the ideal position for thin-film deposition. The layer thickness has been measured at two distinct *y* positions on different silicon substrates to determine how the thickness changes with distance in the vertical *y* direction. This ratio *m* (the ratio of layer thickness to the distance) describes a slope, *i.e.* a change in thickness, due to imperfect positioning of the substrate. To give an example, a B_4_C layer was prepared at +5.5° and a difference of 2.8 nm in layer thickness was measured over 60 mm. Then, accordingly the slope is positive with *m* = 4.6 × 10^−8^. The trend in experimental results is linear, as expected. At the vertical position near 0°, the *m* value was about 0.02 µrad over ±0.3°. Using this result, it is possible to estimate an error in thickness due to the positioning of a typical substrate, while being coated with boron carbide using the above described geometrical assembly. Assuming that a typical mirror is about 100 mm wide (in the sagittal direction) and that an angle of inclination of ±0.3% is obtainable, the ratio *m* is about 2 × 10^−8^. This conservative estimation indicates a change of coating thickness of less than 2 nm, which fulfils the specifications for upcoming light sources.

## 4. Annealing of B_4_C coatings   

**Figure 6:**
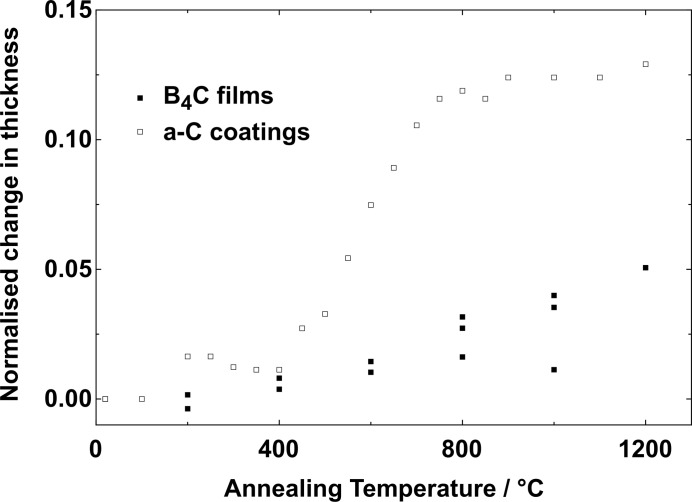
Variation in layer thickness of annealed boron carbide and carbon coatings.

In order to investigate the thermal stability of B_4_C coatings, vacuum annealing experiments were carried out in a stepwise manner up to a temperature of 1200°C. The reflectivity was measured as a function of the incidence angle at each annealing step temperature after 10 min. The layer structure of the B_4_C films was stable up to 1200°C. At a temperature of 1200°C the film peeled off the substrate; this is most likely due to a change in surface structure of the sapphire substrate or a loss of adhesion between the film and the substrate. In Fig. 6 ▸, the normalized layer thickness is compared with previous results for amorphous carbon coatings (Jacobi *et al.*, 2002 ▸). The thickness of the B_4_C layer increases slightly (by less than 4%) during annealing. The increase in thickness was 3× larger in the case of a-C coatings and was explained by the graphitization of carbon. It can be concluded that the thermal stability of B_4_C is much higher than that of a-C coatings. It is, therefore, expected that B_4_C will be a suitable thin-film coating material for FEL mirror applications. Another important material property of B_4_C thin films is their internal compressive stress (Soufli *et al.*, 2009 ▸), which is caused by atomic shot-peening during the deposition process (D’Heurle, 1970 ▸). After coating with a 50 nm-thick layer of B_4_C a thin Si-wafer substrate is significantly bent, whereas a 10 mm-thick Si blank remained unchanged. An investigation is planned to look at this important aspect in more detail using well characterized substrates.

## 5. Surface morphology of B_4_C coatings   

**Figure 7:**
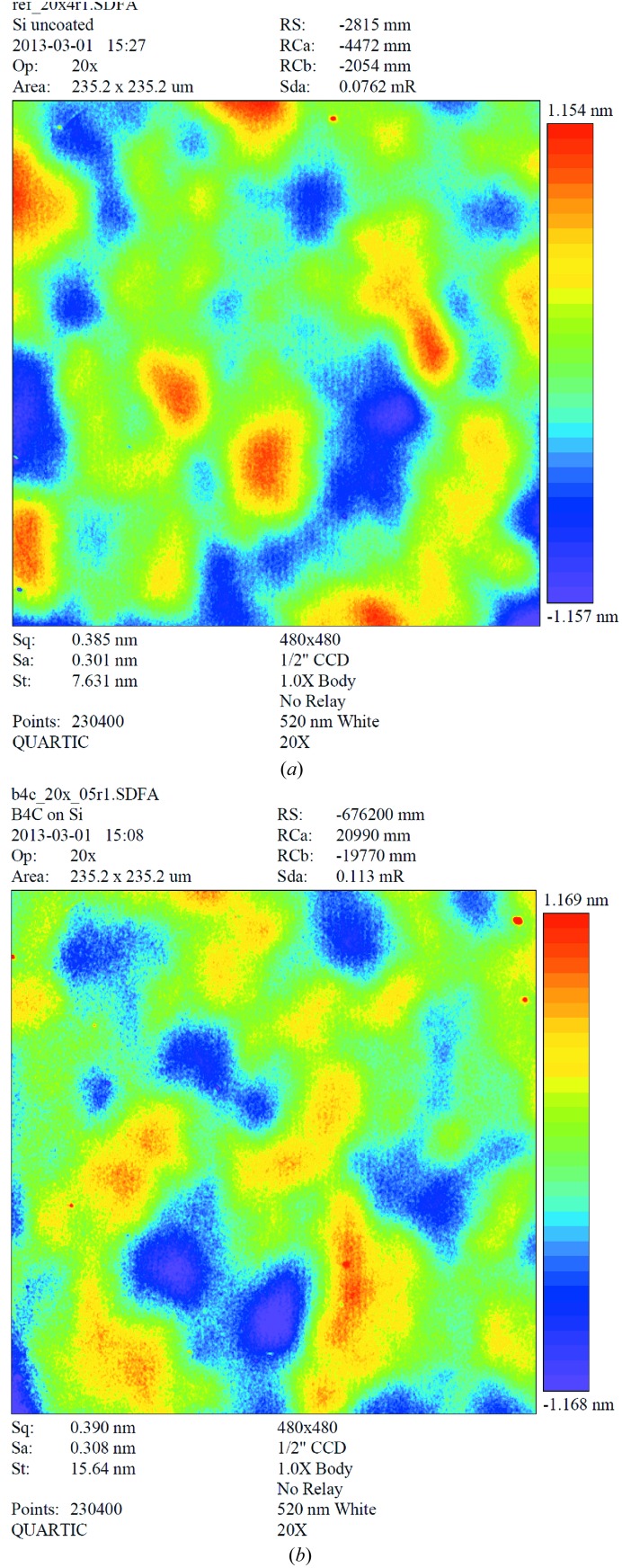
Surface images obtained using optical interferometry with a magnification of ×20: (*a*) uncoated silicon substrate and (*b*) magnetron-sputtered B_4_C coating on a Si substrate.

**Figure 8:**
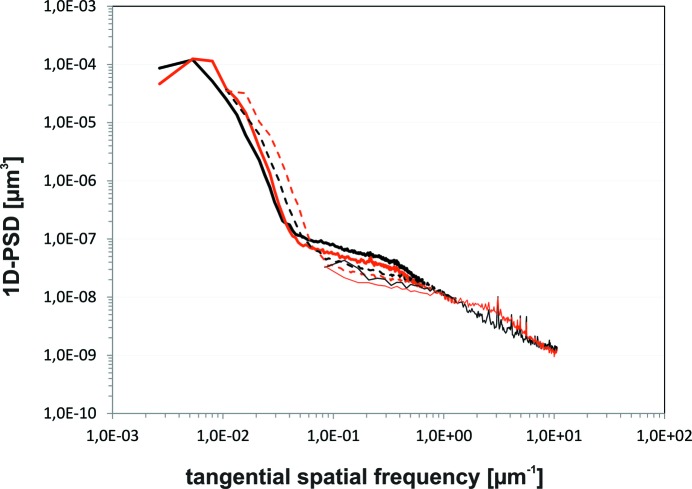
One-dimensional PSD function of a B_4_C coating on silicon.

The microroughness of several boron carbide coatings was investigated using two methods covering a broad range of spatial frequencies from 0.001 to 10 µm^−1^. The uncoated and coated areas of well polished substrates (silicon and sapphire) were investigated using AFM. Furthermore, the microroughness values for the B_4_C coatings were studied at a magnification of 20× using a Micromap Promap 512 white-light interferometer (WLI). Fig. 7 ▸ shows two surface images, one of the uncoated sapphire substrate and the other of a 45 nm-thick B_4_C coating on a sapphire substrate. The aim was to measure different spatial frequency ranges and then interpolate between the measurements using a one-dimensional power spectral density (PSD) function (Fig. 8 ▸). The mid spatial frequency (MSF) part of the one-dimensional PSD function was averaged over five or six measurements using a WLI. One measurement was made in the high spatial frequency (HSF) range on a 15 µm × 15 µm area using AFM. The results are listed in Table 2 ▸. At a HSF, the roughness is 0.12 nm r.m.s. before and after coating. In the MSF range, the microroughness values are 0.18–0.26 nm r.m.s. and 0.35–0.47 nm r.m.s. at magnifications of 40× and 20×, respectively. It can be concluded that there is no important difference in microroughness before and after coating and that the surface quality of the film is predetermined by the surface quality of the substrate.

## 6. Outlook and conclusions   

It has been shown that the challenging specifications for long X-ray mirrors for upcoming free-electron lasers can be achieved; in particular, the peak-to-valley (PV) shape error can be maintained below 2 nm along the optical aperture of about 1 m length. Boron carbide (B_4_C) is a very promising coating material because it is expected that it will withstand single-shot damage at the European XFEL. Using the HZG sputtering facility which has a deposition length of 1500 mm, several B_4_C coatings were manufactured to investigate and characterize the coating precision, stability and repeatability of the coating process and its products. The thickness uniformity over the whole coated area has been determined using X-ray reflectometry. The substrate carrier movement along the long mirror axis (*i.e.* in the tangential direction) has been precisely adjusted to achieve a near constant coating thickness. A waisted mask in front of the sputtering source has been designed and used to reduce thickness variations along the small mirror axes (sagittal direction). The thickness variations achieved were 0.5 nm PV (at a mean thickness of 47.6 nm) and 2.0 nm PV in the tangential and the sagittal directions, respectively. In future mirror production for the European XFEL project, the substrate will be centred on the carrier to avoid edge effects resulting from the sputtering profile and to achieve a coating thickness variation below 1 nm PV. It has also been demonstrated that the influence of a small substrate inclination on the variation of thickness can be eliminated. It is possible to control the substrate inclination to better than ±0.3°. The FEL beam will be totally reflected by the X-ray mirrors below a critical angle that is proportional to the square-root of the film density. Typical layers of magnetron-sputtered B_4_C have a density of 2.37 g cm^−3^, which is about 94% of the bulk value. The thermal stability of B_4_C layers has also been investigated. During annealing, there is a slight increase in layer thickness of up to 4%, which most likely can be explained by relaxation processes. After annealing at 1000°C, the films remain amorphous and stable. At 1200°C, a loss of adhesion between the substrate and the film takes place as the whole film peels off. The microroughness of B_4_C coatings on silicon and sapphire substrates was investigated in the high and mid spatial frequency ranges by optical interferometry and atomic force microscopy. After comparing the surfaces of uncoated and coated areas, it can be stated that the B_4_C coating does not change the surface roughness over any part of the spatial frequency range investigated. This indicates that magnetron-sputtered coating replicates the surface roughness of the substrate. To summarize, the experimental results for B_4_C coatings showed excellent thickness uniformity, high density, excellent thermal stability and low roughness, *i.e.* typical properties of thin films produced using magnetron sputtering.

In future work it is planned to use various materials to coat very long (up to 1.5 m) silicon substrates for use in applications such as plane mirrors, benders and gratings for advanced research light sources, especially the European XFEL project. For total reflection in the hard X-ray regime at a wavelength of 0.1 nm, a mirror length of 1000 mm is required to distribute the expected very high peak power of the XFEL to avoid single-shot damage and to preserve the wavefront of the FEL beam. The substrate surface finishing should have extraordinarily high surface properties characterized by a slope error of 20–50 nrad r.m.s. (equivalent to a radius larger than 6000 km) over a 1 m-long mirror. A second challenge is to coat these optical elements without changing the shape error of 2 nm PV over the whole optical aperture of the mirror. Both requirements are very demanding and have not been met so far over such large areas. Moreover, it is currently not possible to measure such a high surface quality with any currently available methods. In the near future, a first very large prototype will be manufactured, analysed, coated and then characterized. The HZG coating facility is capable of coating mirrors for the most scientifically challenging applications at X-ray sources such as the European XFEL and other advanced research light sources.

## Figures and Tables

**Table 1:** Properties of magnetron-sputtered B_4_C coatings, together with typical data for three metallic mirror materials The properties were determined using Cu radiation (8048 eV) and the coatings deposited onto standard silicon wafer substrates. It should be noted that the surface roughness of a typical silicon substrate used for calibration is similar to that of the later deposited coatings. The layer roughness values were determined by means of X-ray reflectometry and model calculations. Additional roughness values are given in Table 2. ▸

Coating material	Layer thickness (nm)	Layer density (g cm^−3^)	Layer roughness (nm)	Critical angle (°)
				Experimental/theoretical
B_4_C	58.8	2.37	0.42	0.22/0.22
Mo	4.8	10	0.36	0.35/0.43
Rh	45.3	12	0.39	0.47/0.48
Au	10.7	19	0.36	0.55/0.56

**Table 2:** Microroughness values of uncoated and B_4_C coated sapphire substrates Values measured by white-light interferometry (WLI) and atomic force microscopy (AFM) at HZB.

Magnification		Uncoated sapphire	B_4_C coating
WLI 20×	*S*_q_	0.35–0.42 (nm) r.m.s.	0.33–0.47 (nm) r.m.s.
WLI 40×	*S*_q_	0.18–0.26 (nm) r.m.s.	0.20–0.26 (nm) r.m.s.
AFM 15 × 15 (µm)	*S*_q_	0.12 (nm) r.m.s.	0.10 (nm) r.m.s.
